# Differential Growth Responses to Water Balance of Coexisting Deciduous Tree Species Are Linked to Wood Density in a Bolivian Tropical Dry Forest

**DOI:** 10.1371/journal.pone.0073855

**Published:** 2013-10-07

**Authors:** Hooz A. Mendivelso, J. Julio Camarero, Oriol Royo Obregón, Emilia Gutiérrez, Marisol Toledo

**Affiliations:** 1 Instituto Boliviano de Investigación Forestal (IBIF), Universidad Autónoma Gabriel René Moreno, Santa Cruz de la Sierra, Bolivia; 2 Instituto Pirenaico de Ecología (CSIC), Zaragoza, Spain; 3 ARAID, Instituto Pirenaico de Ecología (CSIC), Zaragoza, Spain; 4 Departament d'Ecologia, Universitat de Barcelona, Barcelona, Spain; University of California, Berkeley, United States of America

## Abstract

A seasonal period of water deficit characterizes tropical dry forests (TDFs). There, sympatric tree species exhibit a diversity of growth rates, functional traits, and responses to drought, suggesting that each species may possess different strategies to grow under different conditions of water availability. The evaluation of the long-term growth responses to changes in the soil water balance should provide an understanding of how and when coexisting tree species respond to water deficit in TDFs. Furthermore, such differential growth responses may be linked to functional traits related to water storage and conductance. We used dendrochronology and climate data to retrospectively assess how the radial growth of seven coexisting deciduous tree species responded to the seasonal soil water balance in a Bolivian TDF. Linear mixed-effects models were used to quantify the relationships between basal area increment and seasonal water balance. We related these relationships with wood density and sapwood production to assess if they affect the growth responses to climate. The growth of all species responded positively to water balance during the wet season, but such responses differed among species as a function of their wood density. For instance, species with a strong growth response to water availability averaged a low wood density which may facilitate the storage of water in the stem. By contrast, species with very dense wood were those whose growth was less sensitive to water availability. Coexisting tree species thus show differential growth responses to changes in soil water balance during the wet season. Our findings also provide a link between wood density, a trait related to the ability of trees to store water in the stem, and wood formation in response to water availability.

## Introduction

Tropical dry forests (TDFs) cover almost 17% of the Earth's tropical landmass and constitute one of the most diverse forest ecosystems in the world [1], [2]. TDFs are also among the most threatened lowland tropical forests types, with habitat loss mainly caused by human-induced forest removal due to logging and farming [3]. Therefore we need a better knowledge of the main climatic factors driving forest recovery and tree growth in TDFs to inform the conservation of the forests that remain.

The existence of a dry season, which may last from two up to seven months, causes sharp temporal and spatial changes in soil water balance and tree phenology [4], [5], [6]. In TDFs many tree species are deciduous for two to four months during the dry season [7]. Leaf phenology has also been linked to contrasting growth rates and water transport efficiency [8], [9], [10]. Such varied patterns in aboveground phenology are also linked to the cambium activity through the tree water status which mainly depends on water uptake through the root system, water loss though the leaves, and water storage in the wood [4]. The variations in the leafless periods of TDF tree species have been also correlated with different functional features, such as wood density and water content, which could explain diverse responses to drought and contrasting growth responses to the soil water balance in TDFs [11], [12]. These observations suggest that the seasonal changes in the soil water balance may act as the main driver of tree growth in TDFs.

In TDFs coexisting tree species may show differential growth responses to the seasonal changes in the soil water balance, but within-species variation also provides fundamental information to understand how growth responds to climate [13]. In fact, variability levels in species' traits, both inter-specific (*e.g.*, wood hydraulic properties; cf. Markesteijn *et al.* [12]) and intra-specific (*e.g.*, temporal autocorrelation in radial growth; cf. Zuidema *et al.* [14]), explain different responses to the changing water balance in TDFs. The seasonality imposed by a dry period causes an annual rhythm in cambial activity, and this leads to the formation of annual tree rings in many tree species [15]. Therefore, TDFs constitute a good study system to evaluate how coexisting tree species respond to varying levels of the soil water balance by showing differential growth responses. These responses represent long-term growth information recorded in annual tree-ring widths, which can be recovered using dendrochronology methods [16].

Here, we aim to determine how coexisting deciduous tree species respond to the soil water balance in TDFs by analyzing long-term climate-growth relationships. We studied seven tree species in a TDF located in the Chiquitana region of eastern Bolivia, where most tree species shed their leaves in the dry season [17]. We hypothesize that coexisting tree species will show contrasting growth patterns and differential responses to the soil water balance linked to different traits (*e.g.*, leafless period duration), particularly those related to their ability to store water in the stem (*e.g.*, wood density, sapwood production).

## Materials and Methods

### Ethics Statements

All field sampling was carried out with permission from the national authority (Autoridad Bosques y Tierra, Bolivia). Wood samples were collected from previously felled trees.

### Study site

**Figure 1:**
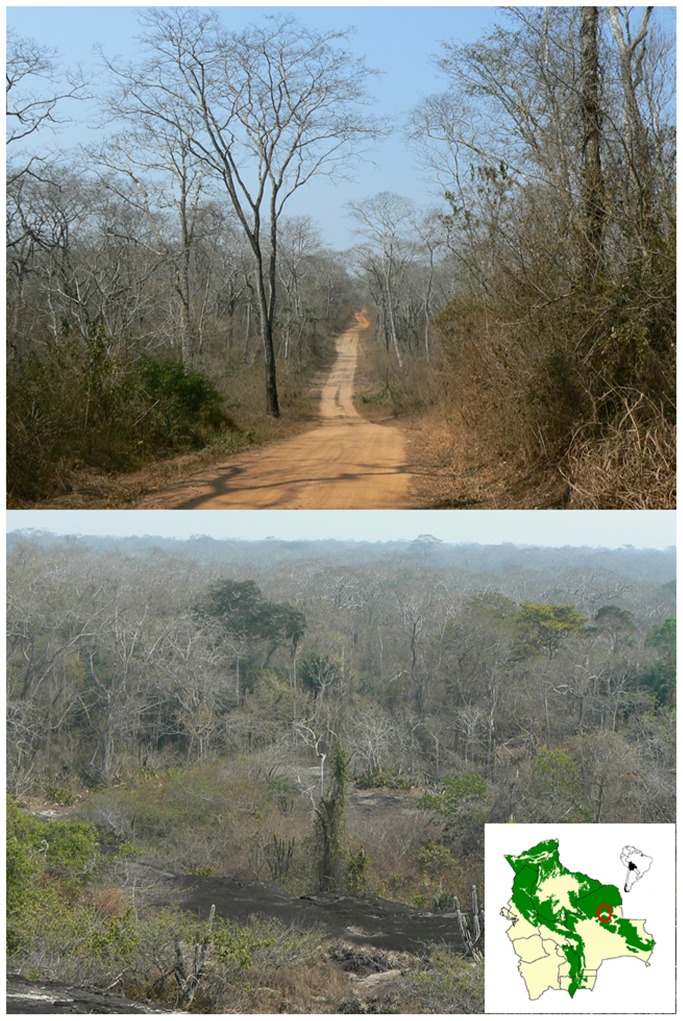
Views of the Bolivian Chiquitano tropical dry forest during the dry season. The inset shows the location of the study site (circle) within Bolivia (green areas correspond to forests).

The study site is a seasonally deciduous lowland TDF located 32 km from Concepción (department of Santa Cruz) in eastern Bolivia (Fig. 1). The study site (16°07′S, 61°43′W, mean elevation of 380 m) is found in a private property of a certified timber company (INPA Parket) and it is included within the network of long-term research sites of the Instituto Boliviano de Investigación Forestal (IBIF; see http://www.ibifbolivia.org.bo/index.php/Monitoreo/INPA). The study site belongs to the Chiquitano forest formation, situated on the Precambrian Brazilian shield, and is considered one of the largest (occupying ca. 16.4 millions of ha.) and most diverse TDFs in Bolivia. Chiquitano forests are located in the transition of the Amazonian lowland evergreen rain forest in the north and the Chaco shrub dry formations towards the south [18]. The studied forest has an average tree richness of 34 species ha^−1^, mean stem density of 437 trees ha^−1^, basal area of 19.7 m^2^ ha^−1^, average canopy height between 20 and 25 m, and 19 out of the 59 tree species have timber value [17], [19]. In the study area the soils are acid (pH = 5.8), contain low organic matter contents (1.42%), and have a sandy-loam texture.

**Figure 2:**
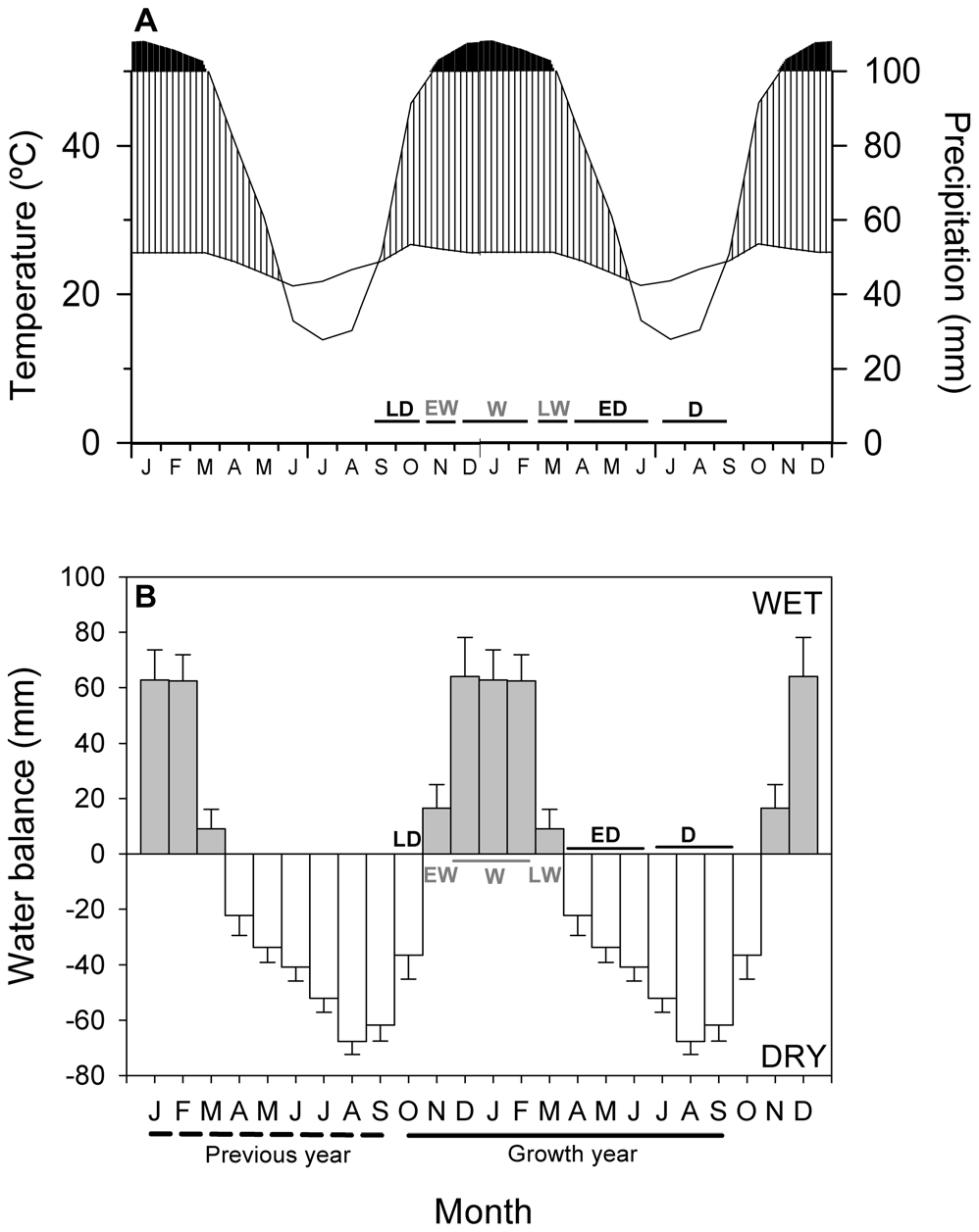
Climatic conditions of the study area. Data from a nearby Concepción meteorological station (A), and estimated monthly water balance (B). The previous (dashed line) and current (continuous line) growth years correspond to the years when the tree ring formation started and ended, respectively. Abbreviations: LD, late dry season; EW, early wet season; W, wet season; LW, late wet season; ED, early dry season; D, dry season. Values of water balance are means ± 1SE.

Meteorological data were obtained from the Concepción station (16°15′S, 62°06′W; period 1949–2009), located about 35 km from the study site. At this station the mean annual temperature was 24.3°C and the recorded total precipitation was 1160 mm. The driest period normally occurs from June to September but the dry season may last up to seven months, from April to October, in which the potential evapotranspiration exceeds precipitation, *i.e.* the water balance is negative (Fig. 2).

### Tree species

We sampled the seven most abundant deciduous tree species forming distinct growth rings (Table 1). All are considered timber tree species, with the exception of *A. cardenasii* which has not been exploited in INPA. Nomenclature of sampled tree species (Table 1) follows the Tropicos webpage [20]. The selected species belong to three different families and display contrasting crown positions, shade tolerances and leafless periods (Table 1). One of the studied species is restricted to the Bolivian Chiquitano TDF (*Acosmium cardenasii*), whereas the others are found in other Bolivian tropical forests (*Aspidosperma tomentosum*, *Centrolobium microchaete*, *Zeyheria tuberculosa*), or are widespread across the Neotropics (*Anadenanthera macrocarpa*, *Caesalpinia pluviosa*, *Tabebuia impetiginosa*).

**Table 1:** Characteristics of the seven tree species studied in the INPA site, a tropical dry forest located in the Chiquitana region of Bolivia.

Family	Species	Crown position	Shade tolerance	Leafless period (months)	Basal area (m^2^ ha^−1^)	Density (stems ha^−1^)
Bignoniaceae	*Zeyheria tuberculosa* (Vell.) Bureau	Canopy	Intolerant	October–November (2)	0.04	2.75
Bignoniaceae	*Tabebuia impetiginosa* (Mart. ex DC.) Standl.	Canopy	Intolerant	July–September (3)	0.27	1.83
Fabaceae	*Centrolobium microchaete* (Mart. ex Benth.) H.C.de Lima *ex* G. P. Lewis	Canopy	Intolerant	June–October (5)	0.55	11.42
Fabaceae	*Anadenanthera macrocarpa* (Benth.) Brenan	Emergent	Intolerant	June–October (5)	2.00	13.67
Fabaceae	*Acosmium cardenasii* H.S. Irwin & Arroyo	Sub-canopy	Tolerant	August–September (2)	6.89	159.25
Fabaceae	*Caesalpinia pluviosa* DC	Canopy	Partially tolerant	August–September (2)	1.18	15.17
Apocynaceae	*Aspidosperma tomentosum* Mart.	Sub-canopy	Partially tolerant	July–September (3)	0.40	12.92

Leaf phenology data were based on field observations and on data presented by Mostacedo [17].

Basal area and density data (considering only stems with diameter at 1.3 m>10 cm) were taken from Mostacedo [17].

### Field sampling

Wood samples (discs) were collected from living and healthy trees felled for timber in 2010 and 2011 and evaluated for growth and sapwood area. Each wood disc was taken at heights ranging from 0.2 to 2.5 m above the ground and discs were always collected above buttresses. In order to reduce spatial autocorrelation in growth patterns, we selected trees that were more than 20 m apart. Additionally, for each species we collected radial cores including sapwood (5-mm wide, 30-mm long) from five trees per species using a Pressler increment borer. The sampled individuals were located in the same stands where the wood discs were collected.

### Wood density and sapwood area

To estimate the potential water storage capacity of the stems we calculated wood density (D) and sapwood area using radial cores and wood discs, respectively. The volume of the radial core (V) was estimated as the volume of a perfect cylinder. The radial cores were oven-dried at 60°C for 48 hours and weighed to obtain the dry weight (DW). Wood density (in g cm^−3^) was calculated according to Borchert [4] as follows:
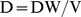
(1)


In the laboratory, the wood discs (*n* = 63) were air-dried and sanded using successively finer grained sandpapers up to 600 grit until the tree rings were clearly visible. A digital picture of each wood disc was taken to calculate the relative stem wood area occupied by sapwood using the ImageJ image analysis software [21]. The distinction between heartwood and sapwood was visually determined by colour differences, which were very evident in five species (*A. macrocarpa*, *C. microchaete*, *C. pluviosa*, *T. impetiginosa*, and *Z. tuberculosa*; see Fig. S1), and the other two species (*A. tomentosum* and *A. cardenasii*) were checked by applying bromocresol green stain on the wood [22]. We assumed that the contraction of dried sapwood in the laboratory would be similar among the seven tree species. All measured samples corresponded to wood discs with areas varying between 1000 and 2000 cm^2^. This size range is similar to those of previous analyses on sapwood area in tropical tree species [23].

### Dendrochronological methods

Tree-ring series were visually cross-dated by comparing and detecting characteristic rings among radii of the same tree and then by matching the series from different trees of the same species. We measured the ring widths from the most recent ring up to the pith along three to four radii per disc, separated by 90–120° to account for: (*i*) the eccentric growth of most discs and (*ii*) the within-tree (among radii) growth variability (see Fig. S1). Hence, the mean value of the ring widths measured along different radii was considered to represent an average radial-growth series of each tree. We assigned to each ring the calendar year in which ring formation began according to the dating convention for the southern hemisphere [24]. Tree rings were measured under a binocular scope using a LINTAB measuring device (Rinntech, Heidelberg, Germany). Then, the previous visual cross-dating was checked using the COFECHA program which calculates the correlation between individual ring-width series and a master series for each species [25]. Only those trees which had established before 1949 were used in further analyses. For each of the seven species, we successfully cross-dated and measured from 7 to 14 trees (Table 2).

**Table 2:** Characteristics of trees and species sampled in a Bolivian tropical dry forest.

Species	No. trees/No. radii	Diameter at 1.3 m (cm)	Age (years)	Wood density (g cm^−3^)	Sapwood area (%)
*Z. tuberculosa*	7/21	38.17±3.60	112±11	0.61±0.01 (a)	42.63±2.15 (d)
*T. impetiginosa*	7/24	43.29±3.39	97±10	0.74±0.04 (bc)	26.79±1.15 (bc)
*C. microchaete*	14/42	36.03±3.15	89±3	0.59±0.02 (a)	29.70±2.03 (c)
*A. macrocarpa*	8/24	42.06±2.85	112±9	0.71±0.05 (bcd)	25.26±3.42 (bc)
*A. cardenasii*	11/38	29.16±3.90	92±5	0.69±0.02 (bd)	16.58±0.69 (a)
*C. pluviosa*	8/31	46.54±3.74	149±10	0.79±0.01 (c)	23.24±2.42 (b)
*A. tomentosum*	8/24	26.89±4.62	84±5	0.66±0.01 (d)	28.07±1.15 (c)

Values are means ± 1SE. Means sharing a letter were not significantly different using paired Mann-Whitney *U* tests (*P*≤0.05).

### Growth patterns

We used basal area increment (BAI) to describe growth patterns because this variable removes the variation in growth attributable to increasing stem circumference and captures changes in growth better than linear measures such as tree-ring width [26]. BAI series for dominant healthy trees usually show an early suppression phase before a rapid increase and a stable phase once trees reach maturity [27]. BAI was calculated as follows:
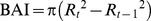
(2)where *R_t_* and *R_t-1_* are the stem radius in years *t* and *t*-1.

Considering the first 70 years of tree life, we calculated BAI trends for each tree as a function of age by using three-parameter logistic functions with information of the following three parameters: maximum BAI (asymptote), maximum growth rate and age when the maximum growth rate is reached. Considering the period 1949–2009, when the maximum number of growth series was available, we also evaluated BAI trends for the mature phase when BAI stabilizes, calculated mean correlations among individual tree BAI series (based on Pearson coefficients), and obtained BAI means and coefficients of variation.

### Definition of the biological growth year and seasonal soil water balances

We defined the biological growth year of the investigated species from October to September (Fig. 2) based on previous phenological studies [17] and dendrometer records of radial increments (Mendivelso & Camarero, *unpublished data*).

We divided the growth year, when tree ring formation starts and ends, in six sub-periods based on their contrasting climatic characteristics and soil water balances (see Fig. 2): (*i*) late dry season (LD, October), (*ii*) early wet season (EW, November), (*iii*) wet season (W, from December to February) when the maximum values of water balance were recorded, (*iv*) late wet season (LW, March), (*v*) early dry season (ED, from April to June), and (*vi*) dry season (D, from July to September) when very low soil water potentials were recorded [6]. We calculated the soil water balance as the difference between the precipitation and the potential evapotranspiration (PET). The PET was calculated from monthly values of minimum, maximum, and mean temperatures using the Hargreaves-Samani method [28].

### Linear mixed-effects models of growth

We fitted linear mixed-effects models to evaluate the relationships between BAI residuals and the six defined climatic sub-periods in the biological growth year (plus the same sub-periods lagged by 1 year) taking into account the variability among individuals (within species) and among species. We calculated BAI residuals as the differences between the observed and the predicted BAI values, where the predicted BAI was obtained by fitting a logistic to growth data as a function of cambial age. We designated both individual tree and biological growth year as random effects and the climatic sub-periods as fixed effects. The response variable was normalized using logarithms, because the distribution of BAI values was left-skewed. We used growth and climate data for the period 1953–2006 (53 years) considering the previous (*t*-1) and current (*t*) years. The fitted model can be summarized as follows:
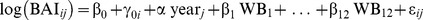
(3)where β_0_ is the intercept, γ_0*i*_ is the random effect related to tree (*i*), α is a parameter adjusting the year (*j*) fixed effect, β_1_… β_12_ are parameters controlling the fixed effects related to water balance (WB) of different sub-periods (WB_1_…WB_12_), and ε*_ij_* is the error term. We accounted for temporal autocorrelation by using an AR(1) correlation structure, *i.e.* by considering a first-order autoregressive model, since BAI usually presents temporal persistence at a one-year lag. We fitted 43 models for each species to evaluate which combinations of climatic variables best explained BAI. Our models ranged from null (only intercept) and single-variable models to more complicated models (see Table S1). The 43 models we fitted constitute only a subset of all possible models, because we excluded unrealistic models and those including highly correlated (*P*≤0.01) pairs of explanatory climatic variables (Table S2). Accordingly, in all of the selected models the variance inflation factor of all fixed factors was <3. The parameters were estimated using the restricted maximum likelihood method [29].

We considered models with substantial support to be those in which we obtained minimum values of the Akaike Information Criterion (AIC), which combines the measure of goodness of fit with a penalty term based on the number of parameters used [30]. We also considered supported models those in which the difference of AIC between the best and following models, *i.e.* those ranked with lowest AIC values, was less than 2. We calculated the Akaike weight (*W_i_*) which is the relative likelihood that the selected *i* model is the best-fitting model for the observed data. The explained variance (R^2^) of the selected models was estimated using a likelihood ratio statistic for each individual tree in all species [31]. Then, we compared the mean R^2^ values among species. The linear mixed-effects models were carried out using the *nlme* package [32] in the R statistical software [33].

## Results

### Growth features of tree species

**Figure 3:**
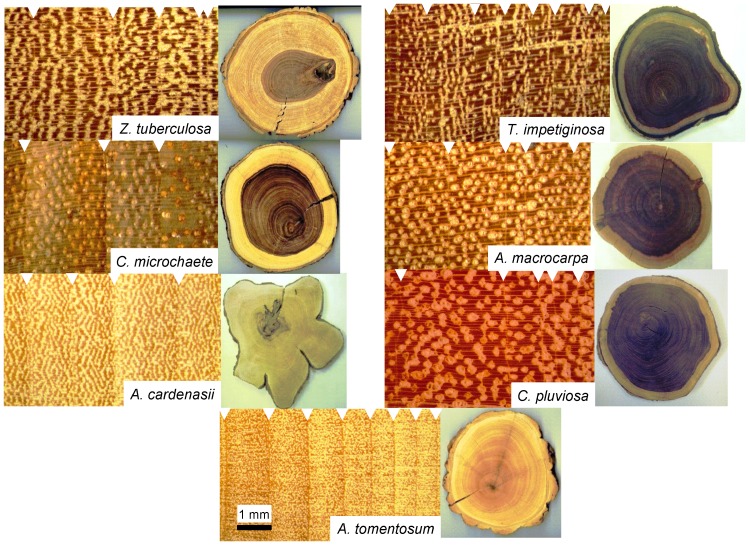
Wood cross-sections with distinct annual rings of studied tree species. The white triangles mark the annual boundaries corresponding to marginal parenchyma. In the rings the growth direction is from left (pith) to right (bark).

The studied species formed diffuse-porous wood and they presented well-defined tree ring boundaries formed by marginal parenchyma (Fig. 3). Wedging rings were sporadically observed in *A. cardenasii* and *C. pluviosa* (Fig. S1). The mean ages of sampled trees varied from 84 (*A. tomentosum*) to 149 years (*C. pluviosa*), whereas the sapwood area reached minimum and maximum values in *A. cardenasii* (13.6%) and *Z. tuberculosa* (42.6%), respectively (Table 2). The wood density varied between 0.59 and 0.79 g cm^−3^. *C. microchaete* and *Z. tuberculosa* exhibited the lowest wood density values while *C. pluviosa* presented the highest.

**Figure 4:**
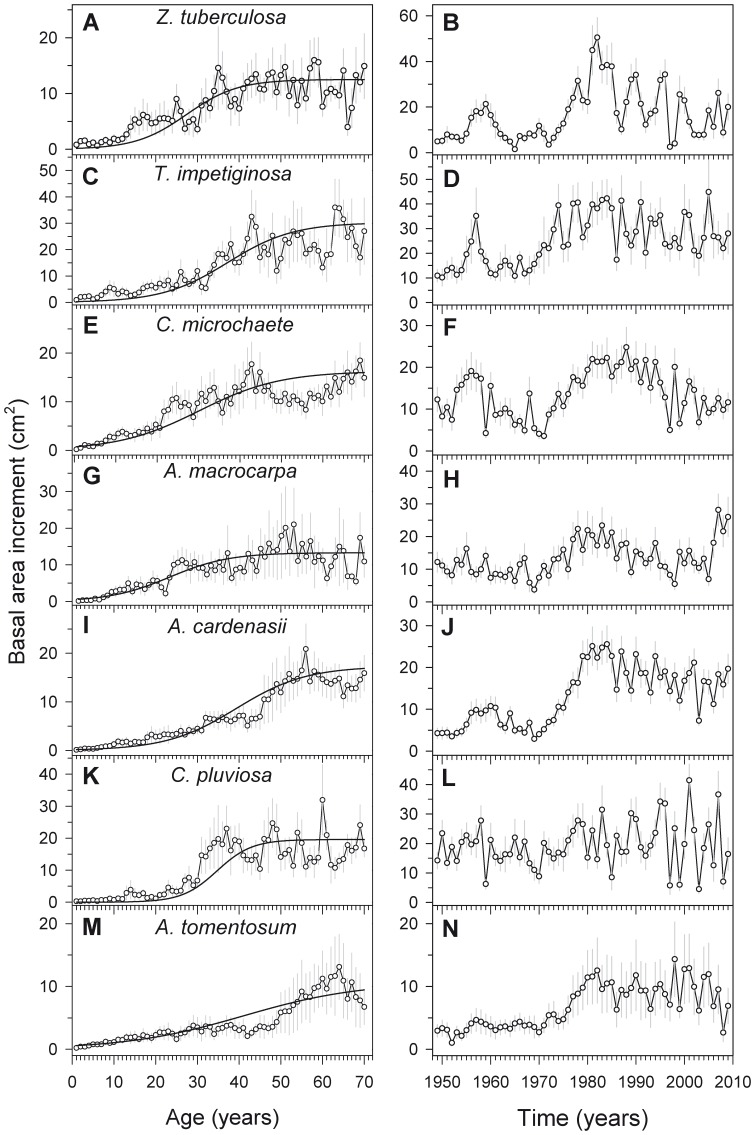
Basal-area increment patterns for the seven tree species from a Bolivian tropical dry forest. Basal area increments (means ± 1SE) are displayed as a function of tree age (A, C, E, G, I, K, M) and calendar year (B, D, F, H, J, L, N). See also the comparisons among tree species displayed in Table 3.

During the first 70 years of life, *A. tomentosum* (a sub-canopy partially shade-tolerant species) and *T. impetiginosa* (a canopy shade-intolerant pioneer species) had the respective lowest (10.7 cm^2^) and highest (30.3 cm^2^) mean BAI values (Table 3; Fig. 4). *A. tomentosum* also showed the lowest BAI rate, whereas the highest rate corresponded to *C. pluviosa* (canopy partially shade-tolerant species). The estimated ages to reach such maximum rate were within a narrow range and varied from 23 (*A. macrocarpa*) to 41 years (*A. tomentosum*).

**Table 3:** Growth-related characteristics of the seven tree species studied in a Bolivian tropical dry forest.

Species	First seventy years of tree life (see Figs. 4 A, C, E, G, I, K, M)			Period 1949–2009 (see Figs. 4 B, D, F, H, J, L, N)		
	Maximum BAI (cm^2^)	Maximum growth rate (cm^2^ yr^−1^)	Age at maximum growth rate (yrs)	BAI (cm^2^)	CV BAI (%)	Correlation between tree BAI series
*Z. tuberculosa*	12.51±1.84 (ab)	1.34±0.47 (b)	27.44±3.72 (ab)	16.41±2.06 (bc)	90.35±5.28 (b)	0.54±0.03 (c)
*T. impetiginosa*	30.26±6.76 (c)	1.59±0.56 (b)	37.28±9.28 (ab)	25.06±3.56 (d)	65.13±3.72 (a)	0.27±0.02 (a)
*C. microchaete*	16.27±3.02 (ab)	0.76±0.20 (b)	30.44±5.88 (ab)	13.63±2.29 (ab)	67.51±2.35 (a)	0.41±0.02 (b)
*A. macrocarpa*	13.29±5.16 (ab)	0.93±0.35 (b)	23.02±5.91 (a)	13.36±2.17 (ab)	73.68±6.82 (ab)	0.23±0.04 (a)
*A. cardenasii*	17.30±3.54 (ab)	1.12±0.62 (b)	41.35±3.99 (b)	13.20±1.87 (ab)	65.99±3.91 (a)	0.62±0.02 (d)
*C. pluviosa*	19.59±2.31 (bc)	2.32±0.75 (c)	34.70±7.18 (ab)	19.34±2.69 (cd)	66.50±3.22 (a)	0.26±0.03 (a)
*A. tomentosum*	10.66±4.47 (a)	0.19±0.08 (a)	39.09±7.85 (b)	6.81±2.60 (a)	63.40±5.46 (a)	0.45±0.04 (b)

Abbreviations note: BAI, Basal area increment; CV, Coefficient of variation. Values are means ± 1SE. Means sharing a letter were not significantly different using Tukey (BAI and growth-rate variables) and Games-Howell (age) *post hoc* tests for those cases with or without homoscedasticity, respectively (*P*≤0.05).

During the common period 1949–2009, *A. tomentosum* (6.8 cm^2^) presented the lowest BAI values while *T. impetiginosa* showed the highest ones (25.1 cm^2^), *i.e.* more than a threefold rise as compared with the former species (Table 3; Fig. 4). *Z. tuberculosa* presented the highest variability in BAI as assessed by the coefficient of variation (90.3%), followed by *A. macrocarpa* (73.7%), while *A. tomentosum* presented the lowest growth variability (63.4%). Finally, the highest within-species coherence among individual BAI tree series (mean correlation values >0.50) was found for *A. cardenasii* followed by *Z. tuberculosa*, while the lowest coherences (mean correlation values <0.30) were observed for *A. macrocarpa*, *C. pluviosa*, and *T. impetiginosa*.

### Relationships between seasonal water variability, BAI, and species traits

**Figure 5:**
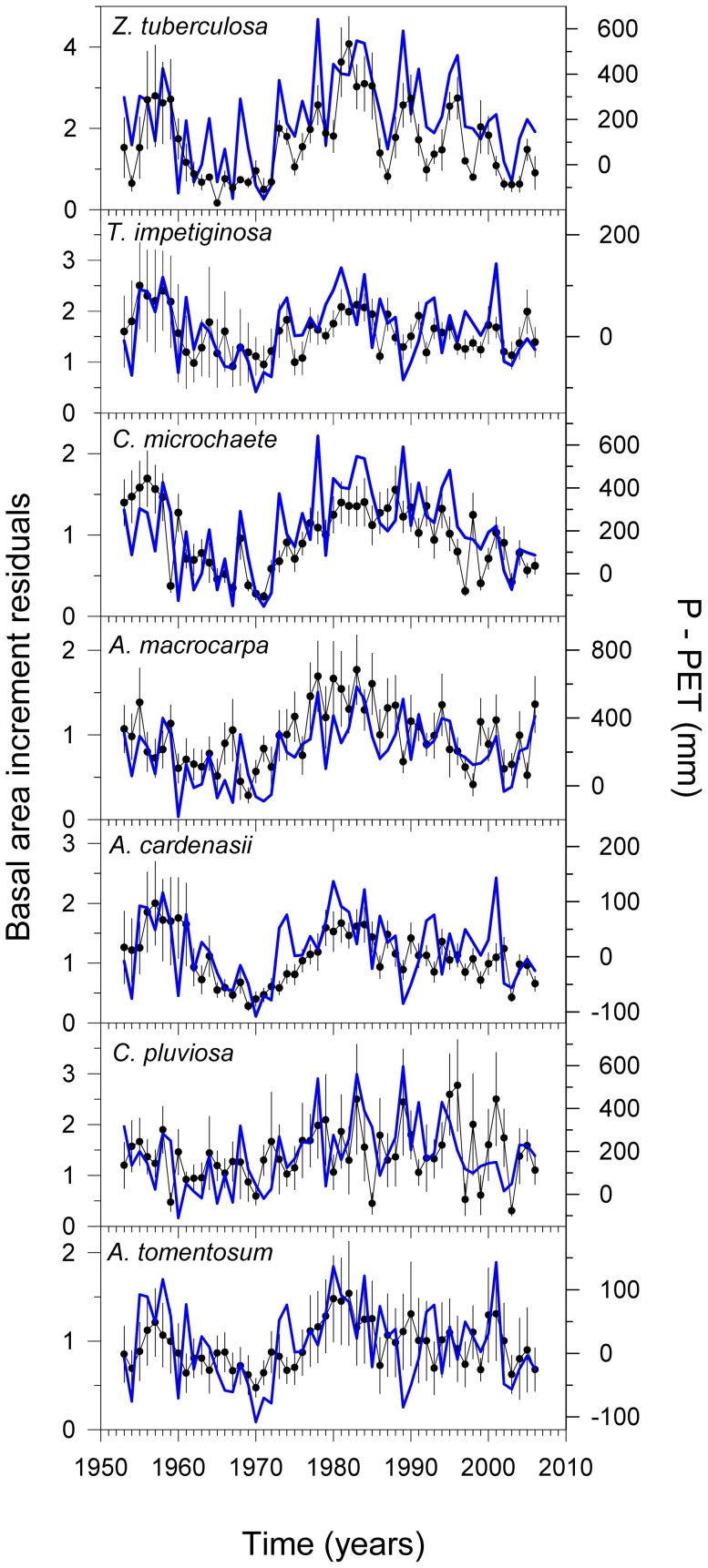
Relationship between basal area increment and soil water balance during the most influential period for growth. Basal area increments (means ± 1SE) residuals and seasonal water balances (those selected as the most influential for tree growth during the current year; see Table 4) are displayed as black and blue lines, respectively. The soil water balance was calculated as the difference between the precipitation (P) and the potential evapotranspiration (PET).

The associations between water balance and BAI were positive for all species during the wet season. However, the species differed in the strength of relationships between growth and water balance during this season (Table 4, Fig. 5; see also Fig. S2). In general, *Z. tuberculosa* was the species that showed the strongest responses to the wettest sub-periods (early wet, wet, and late wet seasons) during the previous and growth years, whereas *A. tomentosum* exhibited moderate responses (early wet season).

**Table 4:** Statistics of the best linear mixed-effects models fitted to basal area increment as a function of soil water balance.

		Previous year						Growth year							
Species	Intercept	LD	EW	W	LW	ED	D	LD	EW	W	LW	ED	D	*Wi*	R^2^
*Z. tuberculosa*	**1.55**		**0.25**	**0.23**	**0.22**				0.14	**0.31**	**0.22**			0.77	0.29±0.05 (a)
*T. impetiginosa*	**1.37**								**0.20**					0.38	0.06±0.01 (b)
*C. microchaete*	**0.96**		**0.09**	**0.08**					**0.13**	**0.09**				0.33	0.19±0.04 (ac)
*A. macrocarpa*	**0.99**								**0.10**	**0.16**				0.79	0.11±0.02 (bc)
*A. cardenasii*	**1.10**		**0.19**						**0.16**					0.96	0.20±0.03 (a)
*C. pluviosa*	**1.43**			**0.14**						**0.12**				0.29	0.06±0.02 (b)
*A. tomentosum*	**0.97**		**0.08**						**0.09**					0.59	0.09±0.04 (bc)

Abbreviations: LD, late dry season; EW, early wet season; W, wet season; LW, late wet season; ED, early dry season; D, dry season. Note the relative probability that the selected model is the best one (*Wi*) and the basal area increment variance (R^2^) explained by the models. Bold coefficients are significant (*P*≤0.05). Means sharing a letter were not significantly different using paired Mann-Whitney *U* tests.

**Figure 6:**
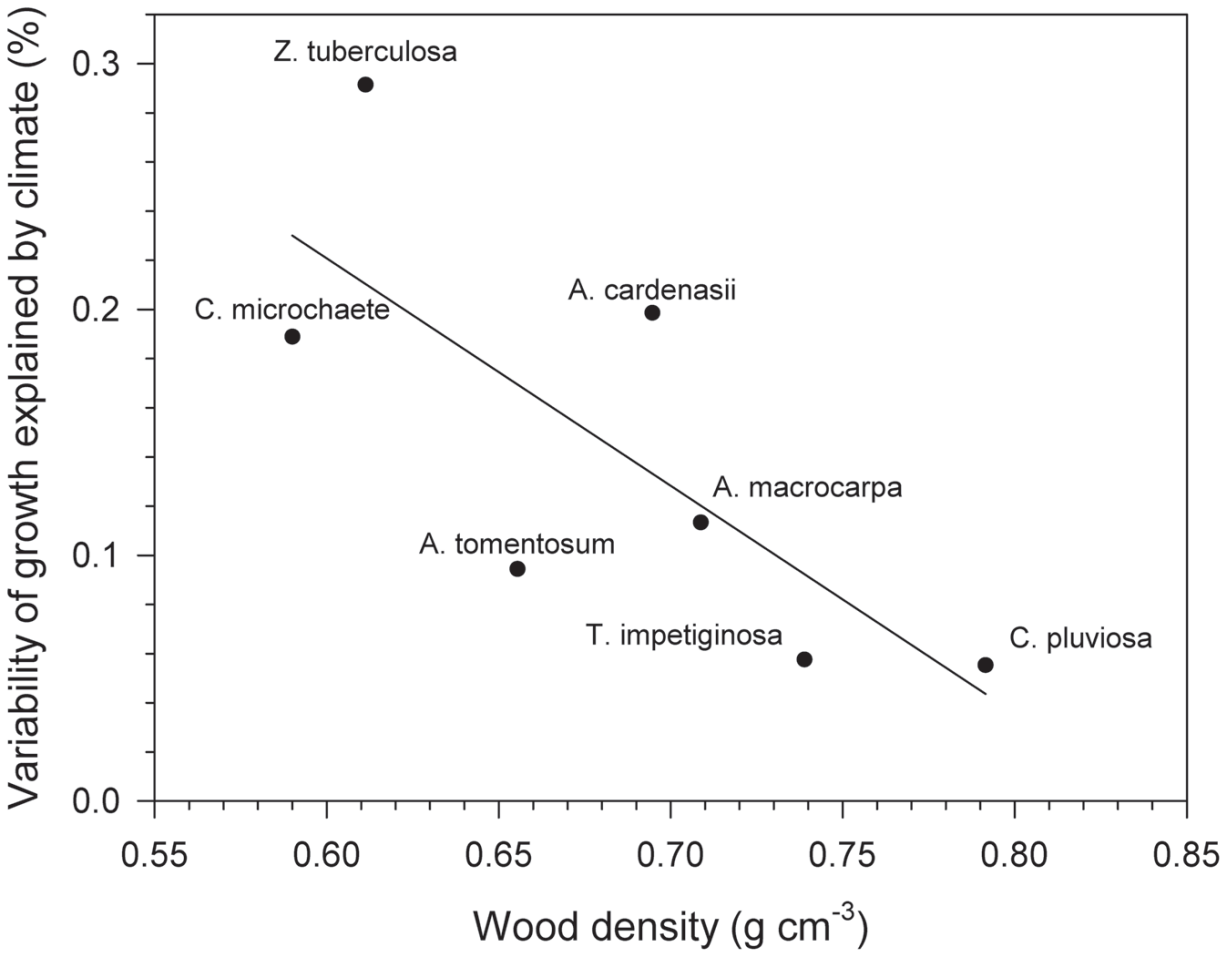
Wood density is negatively related to growth variability driven by water balance. The amount of basal area increment variance (R^2^) explained by the best linear mixed-effects models fitted as a function of soil water balance is shown in the *y* axis (see Table 4).

From the BAI variance explained by the best model fits (see mean R^2^ values, Table 4), the growth of *Z. tuberculosa* (29%) and *A. cardenasii* (20%) was most responsive to water availability, while *C. pluviosa* (6%) and *T. impetiginosa* (6%) were least responsive. The variability of growth explained by water balance was negatively related to wood density (*r* = −0.76, *P* = 0.04) but unrelated to sapwood area (*r* = 0.36, *P* = 0.43). *Zeyheria tuberculosa* was the most sensitive species to water balance and had low wood density values, while, at the other extreme, *C. pluviosa* and *T. impetiginosa* were the least responsive to water balance and had the highest wood density values (Tables 2 and 4, Fig. 6).

## Discussion

### Growth is strongly but differentially related to water balance during the wet season

Our main finding is that radial growth is strongly but differentially related to the wet season water balance in tree species coexisting in a TDF. We discuss the potential mechanisms of such contrasting growth responses to the water balance and how these responses may be linked to functional traits such as wood density.

We found that the studied tree species formed cross-datable annual rings, delimited by marginal parenchyma, a type of parenchyma which is a common anatomical feature in tropical tree species [34]. Such wood anatomy appears to be particularly widespread among deciduous tree species from TDFs [35], [36]. This observation suggests that a pause in cambial activity during the dry season because of low moisture availability probably drives ring boundary formation [37].

The soil water balance is a much stronger driver of tropical tree growth than other factors such as atmospheric CO_2_ concentration, soil type, or disturbance [38], [39]. This finding agrees with other studies from TDFs [40]–[42]. In all studied species, basal area increment increased most during the wettest period of the year, suggesting that the maximum growth rates occur when soil humidity is the highest and probably when carbon uptake also reaches the highest rates [9], [43]. Our findings reveal that growth mainly responds to wet season conditions and species' sensitivty to drought are thus not dependent on dry season conditions, which probably control the late phases of wood formation [37]. However, the variability of growth explained by water balance and maximum juvenile growth rates were not related at all, confirming that these variables are not correlated in the long term.

### Wood density is linked to growth responses to water balance

Based on the best linear-mixed effect models, species were ranked from most to least sensitive according to growth response to water balance,: *Z. tuberculosa*, *A. cardenasii*, *C. microchaete*, *A. macrocarpa*, *A. tomentosum*, *T. impetiginosa*, and lastly *C. pluviosa*. The most responsive species also had a high intra-specific coherence in growth among individuals, possibly mediated by climatic stress. The species with lowest wood density, *i.e.*, a high potential to store water in the sapwood, were those with the highest growth responsiveness to water balance during the wettest sub-periods of both the previous and the year of growth. A good example is *Z. tuberculosa* which showed a strong growth response to water balance during the previous and current-growth years. *Z. tuberculosa* also exhibited a low wood density and a high sapwood area which gives it a higher potential to store water in the stem. Furthermore, this species is able to tolerate drought and keeps its leaves during the driest period. However, there is no relationship between the duration of leaf shedding in the dry season to either wood density or the variability of growth explained by soil water balance when considering all species. Although there was no significant correlation between sapwood area and growth variability explained by the availability of water, *Z. tuberculosa*, the most sensitive species to water balance, exhibited the highest sapwood area (43%), while *C. pluviosa*, the least sensitive species, had one of the lowest sapwood areas (23%). Finally, Fabaceae species (*A. cardenasii*, *A. macrocarpa*, *C. pluviosa*) also had low values of sapwood area which may be related to a strategy for heartwood production in this family [44].

During the short wet season, TDF deciduous tree species maximize photosynthesis, hydraulic conductivity, and growth rate [45], [46]. Deciduous hardwood species with a very dense wood (D>0.8 g cm^−3^) and a low storage ability of water in their stems uptake and deplete water from the shallow soil layers. These species are unable to fully rehydrate their stems after leaf fall during the dry season. Conversely, deciduous softwood tree species usually have less dense wood (D = 0.5–0.8 g cm^−3^) and a high water storage capacity and they reduce water loss during the dry season by leaf shedding [4]. Our study species may be included in this last group which is characterized by a high ability to rehydrate their tissues after the first rainfalls [47]. This fast rehydration ability allows them to quickly reactivate cambial activity [48]. At seasonal scales, the water content of the sapwood and the inner bark also help to explain seasonal changes in girth increment [49]. Lastly, wood density and the ability of deciduous trees from TDFs to store water could explain the mechanisms they use to escape from water stress [50].

Nevertheless, further analyses should consider the multiple roles played by wood density with respect to the water status and the growth responsiveness to climate (hydraulic conductivity, vulnerability to xylem embolism and vessel collapse, water storage) [50]. For instance, tree species with low wood density may also have the largest xylem diameters and thus the highest capacity for water transport and possibly elevated photosynthetic and growth rates under conditions of high water availability, *i.e.* during the wet season. However, in this study, wood density and the maximum growth rates during the first 70 years of life were positively but non-significantly related (*r* = 0.66, *P* = 0.11). Hence, species with denser wood presented the highest juvenile growth rates. This suggests that the increased ability to conduct water provided by a less dense wood likely comes at the expense of a high vulnerability to xylem embolism [50], which requires a tight regulation of stomatal conductance and may reduce growth rates. These potential trade-offs may also contribute to explain the increasing sensitivity of variation of growth rates with decreasing wood densities.

The wide range in wood density of TDF tree species suggests that tree water status and cambium phenology may be related to soil water balance. Perhaps, tree species in TDFs may be able to tap increasingly deeper sources of soil water as the dry season progresses [51]. To test whether stem water storage drives changes in growth responsiveness to water balance we need additional data on the actual water status of trees and how they take up water during the dry and wet seasons [52]. We speculate that contrasting rooting depths of coexisting tree species might explain different time-dependent growth responses to drought. Species with deep roots might access additional sources of soil water as the dry season progresses and thus show growth responses to drought at longer time lags than species with shallow roots.

### Shade tolerance is not related to juvenile growth patterns

Soil water balance seems also to drive growth during the juvenile life stage since shade tolerance was not related at all with growth rates during the first 70 years of life. The maximum growth rates during that life stage were observed in partially shade tolerant and shade-intolerant pioneer species (e.g., *C. pluviosa*), which may grow rapidly to reach the canopy. Conversely, sub-canopy partially shade tolerant species showed the lowest growth rates (e.g., *A. tomentosum*) and, consequently, took more time to attain high growth rates. Overall, the reported studied species presented similar maximum juvenile growth rates which partially agree with results based on repeated measurements of permanent plots reported by Villegas *et al.* [19] for the same forest. Their observations suggested that species partially tolerant to shade show the highest growth rates.

In moist tropical forests shade-intolerant species should grow more rapidly than shade-tolerant ones in light and open conditions [53]. In TDFs growth differences among sub-canopy juveniles persist over longer time than in rainforests due to higher spatial heterogeneity in soil water availability than in moist forests [54]. This reinforces the potential role played by soil water balance as major driver of growth even in early life stages, and could explain the high intra-specific variability observed in basal area increment values during the establishment process of TDF tree species.

### Searching for links between growth and functional traits

Recently, there has been an intense effort to link functional traits and secondary growth in diverse TDFs (*e.g.*, Poorter *et al.* [55]). Growth seems to be related to several functional xylem traits such as wood density [56] or vessel lumen diameter and hydraulic conductance [57]. Hérault *et al.* [58] concluded that stem traits such as wood density and water content largely explained interspecific differences in growth trajectories of tropical tree species. Growth responses to climate may also depend on additional or unmeasured traits such as water-use efficiency or rooting depth. The relative importance of wood density, compared with other traits for controlling the response of growth to water availability, could not be quantified with the available data in this study. Further support for our ideas may come from field studies characterizing seasonal changes in stem and soil water storage and relating them to cambial dynamics. Nonetheless, to the best of our knowledge, this is one of the few studies providing a link between a key functional trait, wood density, and differential growth responsiveness to water balance in TDFs. Finally, we are confident that our approach reduced the effects of several biases which can influence growth estimates: namely, tree ontogeny, size, and competition [59]. First, sampled trees were of similar ages and diameters and they were sampled in the same site from nearby stands subjected to comparable levels of competition between trees. Second, the use of relative basal area increment made it easier to compare the variability in growth, regardless of tree age and size, among conspecific trees [26].

## Conclusions

We found that soil water balance during the wet season strongly and differentially drives radial growth of coexisting tree species in TDFs. The differences in growth explained by water balance varied among species. The strength of the relationship between water balance and growth varied among species as a function of the wood density. *Zeyheria tuberculosa* was the most sensitive species to water balance, exhibited low wood density values, and high sapwood area which is associated with high stem water storage. These traits are likely related to drought tolerance, which might explain its capacity to retain leaves throughout most of the dry season. The species presenting the opposite characteristics (*e.g.*, high wood density) were those whose growth was the least responsive to water balance (*e.g.*, *C. pluviosa*). Our findings thus provide a link between wood density, a proxy of the tree's ability to store water in the stem, and differential growth responses to water balance.

## Supporting Information

Figure S1 **Images of the radii that were cross-dated and measured in** ***A. cardenasii*** **(A) and** ***A. macrocarpa*** **(B).** Note that *A. macrocarpa* shows a clear distinction between sapwood and heartwood. The scale bars correspond to 10 cm.
(DOC)

Figure S2 **Relation of basal area increment residual to water balance for the different species.** The lines show linear regressions (black lines) and related 95% confidence intervals (gray lines). The Pearson correlation coefficients (*r*) between growth and water balance are indicated for each species with its associated probability level (*, *P*<0.05; **P<0.01).
(DOCX)

Table S1 **Comparison of the 43 linear mixed-effects models fitted to basal area increment as a function of several variables related to water balance of the previous and current growth years.** The first reported model is the null one which only considers an intercept. Abbreviations: BAI, basal area increment; *t*, current growth year; *t*-1, previous growth year; LD, late dry season; EW, early wet season; W, wet season; LW, late wet season; ED, early dry season; D, dry season.
(DOCX)

Table S2 **Relationships between the water availability calculated for six sub-periods during the previous and current growth years.** Abbreviations: LD, late dry season; EW, early wet season; W, wet season; LW, late wet season; ED, early dry season; D, dry season. The asterisk indicates high significance levels of Pearson correlation coefficients (*P*≤0.01).
(DOCX)
