# Supplementary material for: Differential Growth Responses to Water Balance of Coexisting Deciduous Tree Species Are Linked to Wood Density in a Bolivian Tropical Dry Forest
Source: PLoS One. 2013 Oct 7;8(10):e73855. doi: 10.1371/journal.pone.0073855 (PMC3792103; doi:10.1371/journal.pone.0073855)
Supplement: Figure S1 — Images of the radii that were cross-dated and measured in A. cardenasii (A) and A. macrocarpa (B). Note that A. macrocarpa shows a clear distinction between sapwood and heartwood. The scale bars correspond to 10 cm. (DOC) [file pone.0073855.s001.doc]

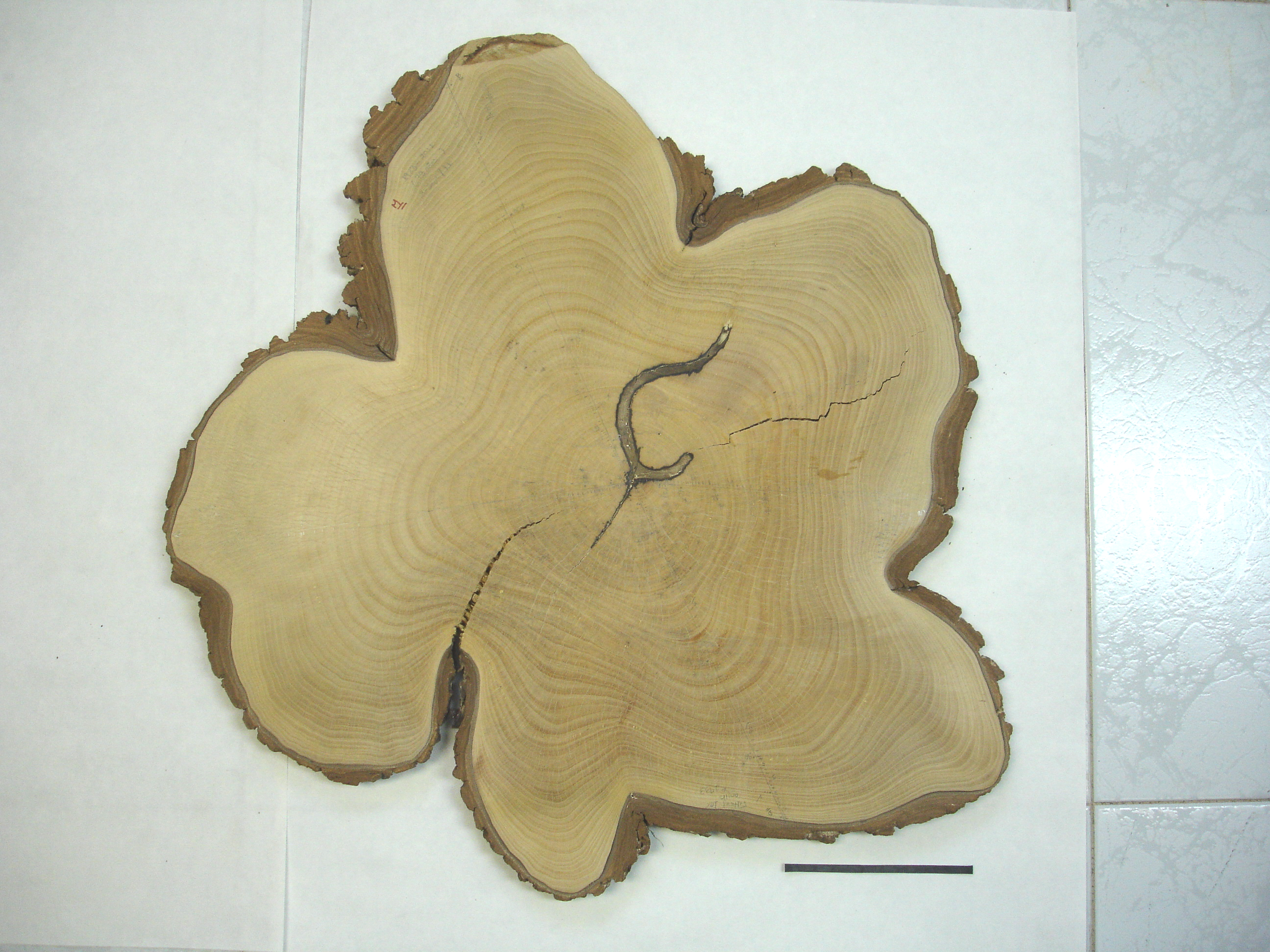

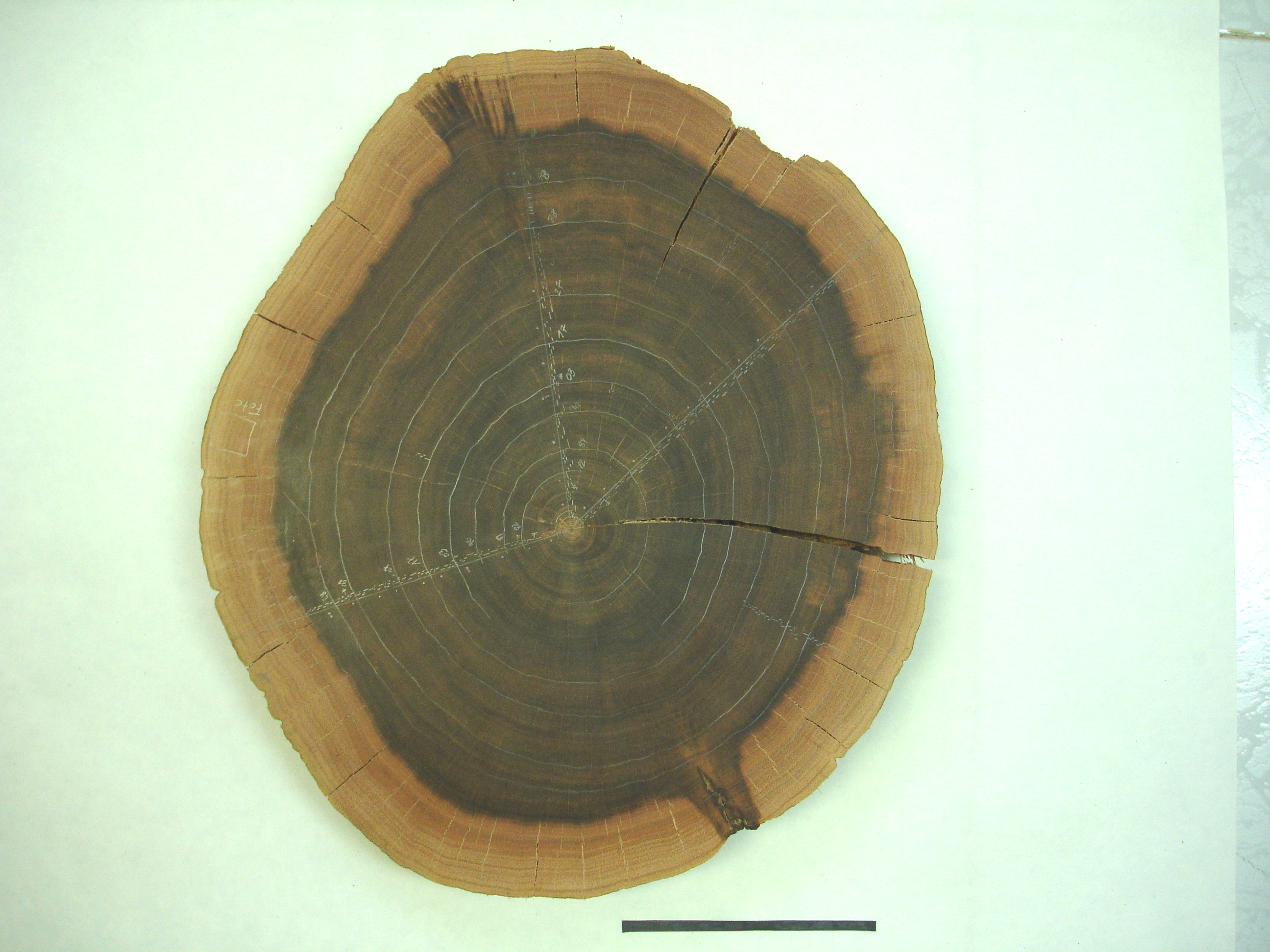


**A**

**B**

**Figure S1.** Images of the cross-dated and measured radii in *A. cardenasii* (A) and *A. macrocarpa* (B) samples.

Note: *A. macrocarpa* shows a clear distinction between sapwood and heartwood. The scale bars correspond to 10 cm.
