# Supplementary material for: Differential Growth Responses to Water Balance of Coexisting Deciduous Tree Species Are Linked to Wood Density in a Bolivian Tropical Dry Forest
Source: PLoS One. 2013 Oct 7;8(10):e73855. doi: 10.1371/journal.pone.0073855 (PMC3792103; doi:10.1371/journal.pone.0073855)
Supplement: Figure S2 — Relation of basal area increment residual to water balance for the different species. The lines show linear regressions (black lines) and related 95% confidence intervals (gray lines). The Pearson correlation coefficients (r) between growth and water balance are indicated for each species with its associated probability level (*, P<0.05; **P<0.01). (DOCX) [file pone.0073855.s002.docx]

**Figure S2.** Relationship between basal area increment residuals and soil water balance for the different species.

The lines show linear regressions (black lines) and 95% confidence intervals (gray lines). The Pearson correlation coefficients (*r*) between both variables are indicated for each species with its associated probability level (*, *P*<0.05; ***P*<0.01).
