# Supplementary material for: Differential Growth Responses to Water Balance of Coexisting Deciduous Tree Species Are Linked to Wood Density in a Bolivian Tropical Dry Forest
Source: PLoS One. 2013 Oct 7;8(10):e73855. doi: 10.1371/journal.pone.0073855 (PMC3792103; doi:10.1371/journal.pone.0073855)
Supplement: Table S1 — Comparison of the 43 linear mixed-effects models fitted to basal area increment as a function of several variables related to water balance of the previous and current growth years. The first reported model is the null one which only considers an intercept. Abbreviations: BAI, basal area increment; t, current growth year; t-1, previous growth year; LD, late dry season; EW, early wet season; W, wet season; LW, late wet season; ED, early dry season; D, dry season. (DOCX) [file pone.0073855.s003.docx]

**Table S1.** Comparison of the 43 linear mixed-effects models fitted to basal area increment as a function of several variables related to water balance of the previous and current growth years.

| **Models** |
| --- |
| BAI ~ β_0_ |
| BAI ~ LD_t_ |
| BAI ~ LD_t-1_ + LD_t_ |
| BAI ~ EW_t_ |
| BAI ~ EW_t-1_ + EW_t_ |
| BAI ~ W_t_ |
| BAI ~ W_t-1_ + W_t_ |
| BAI ~ LW_t_ |
| BAI ~ LW_t-1_ + LW_t_ |
| BAI ~ ED_t_ |
| BAI ~ ED_t-1_ + ED_t_ |
| BAI ~ D_t_ |
| BAI ~ D_t-1_ + D_t_ |
| BAI ~ LD_t_ + EW_t_ |
| BAI ~ LD_t-1_ + EW_t-1_ + LD_t_ + EW_t_ |
| BAI ~ LD_t_ + EW_t_ + W_t_ |
| BAI ~ LD_t-1_ + EW_t-1_ + W_t-1_ + LD_t_ + EW_t_ + W_t_ |
| BAI ~ LD_t_ + EW_t_ + W_t_ + LW_t_ |
| BAI ~ LD_t-1_ + EW_t-1_ + W_t-1_ + LW_t-1_ + LD_t_ + EW_t_ + W_t_ + LW_t_ |
| BAI ~ LD_t_ + EW_t_ + W_t_ + LW_t_ + ED_t_ |
| BAI ~ LD_t-1_ + EW_t-1_ + W_t-1_ + LW_t-1_ + ED_t-1_ + LD_t_ + EW_t_ + W_t_ + LW_t_ + ED_t_ |
| BAI ~ LD_t_ + EW_t_ + W_t_ + LW_t_ + ED_t_ + D_t_ |
| BAI ~ LD_t-1_ + EW_t-1_ + W_t-1_ + LW_t-1_ + ED_t-1_ + D_t-1_ + LD_t_ + EW_t_ + W_t_ + LW_t_ + ED_t_ + D_t_ |
| BAI ~ EW_t_ + W_t_ |
| BAI ~ EW_t-1_ + W_t-1_ + EW_t_ + W_t_ |
| BAI ~ EW_t_ + W_t_ + LW_t_ |
| BAI ~ EW_t-1_ + W_t-1_ + LW_t-1_ + EW_t_ + W_t_ + LW_t_ |
| BAI ~ EW_t_ + W_t_ + LW_t_ + ED_t_ |
| BAI ~ EW_t-1_ + W_t-1_ + LW_t-1_ + ED_t-1_ + EW_t_ + W_t_ + LW_t_ + ED_t_ |
| BAI ~ EW_t_ + W_t_ + LW_t_ + ED_t_ + D_t_ |
| BAI ~ EW_t-1_ + W_t-1_ + LW_t-1_ + ED_t-1_ + D_t-1_ + EW_t_ + W_t_ + LW_t_ + ED_t_ + D_t_ |
| BAI ~ W_t_ + LW_t_ |
| BAI ~ W_t-1_ + LW_t-1_ + W_t_ + LW_t_ |
| BAI ~ W_t_ + LW_t_ + ED_t_ |
| BAI ~ W_t-1_ + LW_t-1_ + ED_t-1_ + W_t_ + LW_t_ + ED_t_ |
| BAI ~ W_t_ + LW_t_ + ED_t_ + D_t_ |
| BAI ~ W_t-1_ + LW_t-1_ + ED_t-1_ + D_t-1_ + W_t_ + LW_t_ + ED_t_ + D_t_ |
| BAI ~ LW_t_ + ED_t_ |
| BAI ~ LW_t-1_ + ED_t-1_ + LW_t_ + ED_t_ |
| BAI ~ LW_t_ + ED_t_ + D_t_ |
| BAI ~ LW_t-1_ + ED_t-1_ + D_t-1_ + LW_t_ + ED_t_ + D_t_ |
| BAI ~ ED_t_ + D_t_ |
| BAI ~ ED_t-1_ + D_t-1_ + ED_t_ + D_t_ |

Note: The first reported model is the null one which only considers an intercept. BAI, to basal area increment; *t,* current growth year; *t*-1, previous growth year; LD, late dry season; EW, early wet season; W, wet season; LW, late wet season; ED, early dry season; D, dry season.
