# Supplementary material for: Differential Growth Responses to Water Balance of Coexisting Deciduous Tree Species Are Linked to Wood Density in a Bolivian Tropical Dry Forest
Source: PLoS One. 2013 Oct 7;8(10):e73855. doi: 10.1371/journal.pone.0073855 (PMC3792103; doi:10.1371/journal.pone.0073855)
Supplement: Table S2 — Relationships between the water availability calculated for six sub-periods during the previous and current growth years. Abbreviations: LD, late dry season; EW, early wet season; W, wet season; LW, late wet season; ED, early dry season; D, dry season. The asterisk indicates high significance levels of Pearson correlation coefficients (P≤0.01). (DOCX) [file pone.0073855.s004.docx]

**Table S2.** Relationships between the water balances calculated for six sub-periods during the previous and current growth years.

|  |  | **Growth year (year *t*)** | | | | | |
| --- | --- | --- | --- | --- | --- | --- | --- |
|  | **Sub-period** | **LD** | **EW** | **W** | **LW** | **ED** | **D** |
| Previous year (year *t*-1) | LD | -0.27* | 0.07 | 0.01 | -0.16 | 0.19 | -0.11 |
|  | EW | -0.07 | 0.29* | 0.15 | 0.23 | 0.23 | -0.01 |
|  | W | -0.05 | 0.10 | 0.25 | 0.31* | 0.18 | 0.23 |
|  | LW | 0.22 | 0.24 | -0.10 | -0.03 | 0.28* | 0.12 |
|  | ED | -0.02 | 0.01 | 0.06 | 0.23 | -0.08 | 0.04 |
|  | D | -0.07 | 0.11 | 0.09 | 0.34* | 0.07 | 0.18 |
| Growth year (year *t*) | LD | −−− | 0.12 | 0.05 | 0.03 | -0.13 | -0.04 |
|  | EW |  | −−− | 0.15 | 0.15 | 0.33* | 0.30* |
|  | W |  |  | −−− | 0.21 | -0.01 | 0.28* |
|  | LW |  |  |  | −−− | 0.13 | 0.15 |
|  | ED |  |  |  |  | −−− | 0.24 |

Note: LD, late dry season; EW, early wet season; W, wet season; LW, late wet season; ED, early dry season; D, dry season. The asterisk indicates high significance levels of Pearson correlation coefficients (*P*≤0.01).
